# Antimicrobial evaluation of plants used for the treatment of diarrhoea in a rural community in northern Maputaland, KwaZulu-Natal, South Africa

**DOI:** 10.1186/s12906-015-0570-2

**Published:** 2015-03-10

**Authors:** Sandy F van Vuuren, Mduduzi N Nkwanyana, Helene de Wet

**Affiliations:** Department of Pharmacy and Pharmacology, Faculty of Health Sciences, University of Witwatersrand, 7 York Road, Parktown, 2193 South Africa; Department of Botany, University of Zululand, Private Bag 1001, KwaDlangezwa, 3886 South Africa

**Keywords:** Antimicrobial, Diarrhoea, Northern Maputaland, Synergy

## Abstract

**Background:**

Zulu people living in the rural area of Maputaland (KwaZulu-Natal, South Africa) rely heavily on medicinal plants for the treatment of diarrhoea. Abundant availability of medicinal plants in the study area offers low cost health care, but scientific validation is needed in order to lend credibility to the traditional use against many ailments including diarrhoeal infections. With this in mind a study was designed to test the *in vitro* antimicrobial efficacy of 23 plant species which are used for the treatment of diarrhoea in rural Maputaland. Four 1:1 plant combinations were also evaluated to determine their interactive effects against seven diarrhoea-related bacterial pathogens.

**Methods:**

Minimum inhibitory concentration (MIC) assays were undertaken on dichloromethane-methanol (CH_2_Cl_2_: MeOH) and aqueous crude extracts. The following micro-organisms were selected for this study and were tested based on their association with stomach ailments and diarrhoea; *Bacillus cereus* (ATCC 11778), *Enterococcus faecali*s (ATCC 29212), *Escherichia coli* (ATCC 8739), *Proteus vulgaris* (ATCC 33420), *Salmonella typhimurium* (ATCC 14028), *Shigella flexneri* (ATCC 25875) and *Staphylococcus aureus* (ATCC 12600). The fractional inhibitory concentration index (ΣFIC) was determined for plants traditionally used in combination.

**Results:**

*Shigella flexneri* proved to be the most susceptible pathogen, where the organic extract of *Terminalia sericea* showed the most prominent noteworthy antibacterial activity (mean MIC value of 0.04 mg/mL). The aqueous extracts generally showed poorer antimicrobial activity with some exceptions i.e. *Acacia burkei, Brachylaena transvaalensis* against *B. cereus* and *B. transvaalensis* against *S. flexneri.* In the combination studies, synergy was predominant with mean (across all pathogens) ΣFIC values of 0.30 for *Acanthospermum glabratum* with *Krauseola mosambicina;* ΣFIC values of 0.46 for *A. glabratum* with *Psidium guajava;* ΣFIC values of 0.39 for *B. transvaalensis* with *P. guajava* and ΣFIC values of 0.88 (additive) for the combination of *B. transvaalensis* with *Sclerocarya birrea.*

**Conclusion:**

This study provided some insight into the bacterial *in vitro* efficacies of plants traditionally used to treat diarrhoea by the people of Northern Maputaland. Very little connection was observed between frequency of use and efficacy. Plant combinations demonstrated favourable efficacy with mostly synergistic effects noted, lending some credibility to their use in combination.

## Background

Diarrhoea is a major medical problem globally and in developing countries like South Africa, it has become prevalent in immunocompromised patients [1,2]. It has been stipulated that between 6.9% of deaths in the low and medium income countries are caused by diarrhoea, making it the third leading cause of death in these countries [3]. Furthermore, infection rates are compounded by the low socio economic climate in countries of Sub-Saharan Africa. In South Africa, for example, more than 25 000 deaths, caused by diarrhoeal diseases, were recorded in 2005 [4].

Northern Maputaland, situated in KwaZulu-Natal, is one of the most poverty stricken areas in South Africa where the availability of clean drinking water and sanitary ablutions are particularly problematic [5]. Under these conditions, diarrhoea is a major concern to resident rural communities and medicinal plants are extensively used to manage and treat these conditions. A review by Njume and Goduka [6], highlights various factors with respect to diarrhoea and medicinal plant use. One aspect of emphasis was that medicinal plants should be readily available and attainable in rural communities. Thus, our previous study documenting the ethnobotanical use of medicinal plants for the treatment of diarrhoea [7] forms a back-bone to further research on efficacy. In our earlier findings, twenty-three plant species were documented as anti-diarrhoeal treatments. Four plants (*Acacia burkei, Brachylaena transvaalensis, Cissampelos hirta* and *Sarcostemma viminale*) were recorded for the first time. The most frequently used plants were three exotic species namely: *Psidium guajava, Catharanthus roseus* and *Melia azedarach* followed by two indigenous species to South Africa namely; *Sclerocarya birrea* and *Strychnos madagascariensis*. Furthermore, several plant combinations were used for antidiarrhoeal efficacy. It thus makes sense to broaden this ethnopharmacological investigation, by determining if these plants, which are readily available and attainable by the residing ethnic populations, are antimicrobially effective against bacterial pathogens responsible for diarrhoea.

## Methods

### Plant collection and extraction

During the ethnobotanical survey [7], plant samples were collected, identified and voucher specimens were deposited in the herbarium at the Department of Botany, University of Zululand. The collected plant samples were dried at ambient temperature and ground into fine powder with a hammer mill. Two types of plants extractions were prepared, a 1:1 mix of dichloromethane:methanol (organic) and an aqueous extract for each plant species. The organic extract was prepared by submerging 10 g of the dried macerated plant material in 100 mL of a 1:1 mixture of dichloromethane and methanol in order to extract for both polar and non-polar compounds. The extract was heated to 30°C for 24 hours. Thereafter it was filtered, evaporated and stored at 4°C. The aqueous extract was prepared to mimic actual preparation in the homesteads. An aqueous extract was prepared by submerging 10 g of the macerated plant material in 100 mL of boiling water, which was then kept at ambient temperature overnight. Thereafter, it was filtered and stored at −80°C before lyophilisation [8].

### Antimicrobial screening

The following micro-organisms were selected for this study based on their association with stomach ailments and diarrhoea and were used in the minimum inhibition concentration (MIC) assay; *Bacillus cereus* (ATCC 11778), *Enterococcus faecalis* (ATCC 29212), *Escherichia coli* (ATCC 8739), *Proteus vulgaris* (ATCC 33420), *Salmonella typhimurium* (ATCC 14028), *Shigella flexneri* (ATCC 25875) and *Staphylococcus aureus* (ATCC 12600). The National Committee for Laboratory Standards [9] as well as Eloff [10] were used as methodology guidelines to determine the MIC. Bacterial cultures were sub-cultured from stock agar plates and grown in Tryptone Soya broth overnight. Microtitre plates were aseptically prepared by adding 100 μL distilled sterile water into each well. Then, 100 μL of the plant extracts at starting stock concentrations of 64 mg/mL were transferred into the microtitre plate. The plant extracts were reconstituted in acetone. Serial dilutions were performed, leading to a final volume of 100 μL per well. The overnight cultures were diluted in fresh Tryptone Soya broth at a 1:100 ratio, yielding an approximate inoculum size of 1 × 10^6^ colony forming units (CFU)/mL. An amount of 100 μL was added to each well. The plates were then covered with sterile adhesive to prevent evaporation of volatile compounds. The plates were then incubated for 24 hours at 37°C. Ciprofloxacin (0.02 mg/mL) was used as a positive control while acetone (64 mg/mL) was used as a negative control. After 24 hours, 40 μL of 0.2 mg/mL of *p*-iodonitrotetrazolium (INT) violet (Sigma) was added into all wells of the microtitre plates. The plates were then kept for six hours at ambient temperature before inspection for antibacterial activity. The INT was used as the bacterial growth inhibition indicator whereby the pink, purple or red colour represented bacterial growth while no colour change represented growth inhibition. The lowest concentration at which the plant extract inhibited bacterial growth was considered as the MIC value for the crude extracts.

As selected plant combinations are used traditionally for the treatment of diarrhoea, these plants were investigated for their interactive efficacies to determine whether efficacy would be enhanced when combined. The MIC method was followed, except 1:1 combinations were prepared from stock solutions (64 mg/mL for extracts with 50 μL of each plant adding up to 100 μL in each well). The MIC value was determined for these combinations. In order to determine the interaction between plants, the fractional inhibitory concentration (FIC) was then calculated using the following equation;

$$ {\mathrm{FIC}}^{\left(\mathrm{a}\right)}=\frac{\mathrm{MIC}\ \left(\mathrm{plant}\ \mathrm{A}\right)\ \mathrm{in}\ \mathrm{combination}\ \mathrm{with}\ \mathrm{plant}\ \mathrm{B}}{\ \mathrm{MIC}\ \mathrm{plant}\ \mathrm{A}\ \mathrm{in}\mathrm{dependently}} $$

$$ {\mathrm{FIC}}^{\left(\mathrm{b}\right)}=\frac{\mathrm{MIC}\ \left(\mathrm{plant}\ \mathrm{B}\right)\ \mathrm{in}\ \mathrm{combination}\ \mathrm{with}\ \mathrm{plant}\ \mathrm{A}}{\mathrm{MIC}\ \mathrm{plant}\ \mathrm{B}\ \mathrm{in}\mathrm{dependently}} $$

The FIC index is determined where ΣFIC = FIC^(a)^ + FIC^(b)^ [11]. The ΣFIC was used to determine the correlation between the two plants and may be classified as either synergistic (≤0.5), additive (>0.5-1.0), indifferent (>1.0- < 4.0) or antagonistic (≥4.0) [12]. Conventional antimicrobials were included in all repetitions and the study was undertaken in triplicate.

## Results and discussion

The antimicrobial efficacy of plant extracts were analysed using Gibbons [13], Rios and Recio [14] and Van Vuuren [15] where criteria stipulated that MIC values <1.00 mg/mL are considered noteworthy. The 23 plant species (organic and aqueous extracts) (Table 1) demonstrated some antibacterial activity with *S. flexneri* being the most susceptible pathogen with efficacies lower than 1 mg/mL for 16 organic plant extracts and two aqueous extracts. *S. flexneri* is a highly infectious Gram-negative pathogen associated with diarrhoea in developing countries where there is a lack of clean drinking water, poor sanitation and malnutrition [16]. Thus, this pathogen showing susceptibility to many of the plants tested, may be controlled to some extent, where lack of clean water and infrastructure is clearly linked to increased infection rates. The most antimicrobially effective plant against *S. flexneri* was *Terminalia sericea,* being highly active both with the organic extract (0.04 mg/mL) and aqueous extract (0.67 mg/mL). The antimicrobial activity of *T. sericea* has been well studied [17-21]. However, other diarrhoeal pathogens have been neglected, particularly studies against *Shigella* spp. This is surprising considering that the traditional use of the plant includes stomach ailments [22]. Furthermore, the highly active antimicrobial effects noted against *S. flexneri* are worthy of highlighting.

**Table 1:** **The antibacterial (MIC values in mg/mL) efficacy of plants used as remedies for the treatment of diarrhoea in northern Maputaland, KwaZulu-Natal, South Africa**

**Plant species, family and voucher number**	**Plant part**	**Solvent**	***B. cereus*** **(ATCC 11778)**	***E. faecalis*** **(ATCC 29212)**	***S. aureus*** **(ATCC 12600)**	***E. coli*** **(ATCC 8739)**	***P. vulgarius*** **(ATCC 33420)**	***S. typhimurium*** **(ATCC 14028)**	***S. flexneri*** **(ATCC 25875)**	**Average MIC**
*Acacia burkei* Fabaceae Benth (MNN^a^-3)	Bark	Organic	3.00	2.00	1.00	1.00	**0.50**	3.00	**0.25**	1.54
		Aqueous	**0.75**	1.00	1.50	1.50	3.00	3.00	1.00	1.68
*Acanthospermum glabratum* Asteraceae (DC) Wild (MNN-37)	Whole plant	Organic	**0.88**	4.00	4.13	6.00	8.00	2.25	**0.44**	3.67
		Aqueous	5.33	NS^b^	6.67	NS	NS	6.00	4.00	5.50
*Brachylaena Transvaalensis* Asteraceae E. Phillips & Schweick. (MNN-30)	Leaves	Organic	NS	NS	NS	NS	NS	NS	4.00	4.00
		Aqueous	**0.25**	1.07	1.25	2.00	1.75	1.25	**0.50**	1.15
*Catharanthus roseus* Apocynaceae (L.) G. Don. (MNN-7)	Roots	Organic	**0.64**	1.93	3.50	3.71	4.07	4.71	**0.41**	2.71
		Aqueous	6.00	6.00	8.00	4.00	4.00	4.00	4.00	5.33
*Chenopodium ambrosioides* Chenopodiaceae L. (MNN-39)	Whole plant	Organic	12.00	6.00	**0.25**	3.00	**0.25**	4.00	**0.50**	3.71
		Aqueous	3.00	8.00	4.00	8.00	3.00	8.00	8.00	6.00
*Cissampelos hirta* Menispermaceae Klotzch (MNN-27)	Whole plant	Organic	2.00	**0.42**	1.50	8.00	2.00	1.33	**0.38**	2.23
		Aqueous	6.00	4.00	6.00	8.00	8.00	8.00	8.00	6.86
*Garcinia livingstonei* Clusiaceae T. Anderson (MNN-19)	Bark	Organic	**0.12**	**0.34**	**0.26**	3.38	**0.75**	**0.19**	**0.38**	**0.77**
		Aqueous	1.50	2.00	**0.75**	**0.75**	1.50	2.00	2.00	1.50
*Gymnosporia senegalensis* Celastraceae (Lam.) Loes. (MNN-13)	Leaves	Organic	**0.94**	**0.56**	2.17	6.00	11.34	**0.79**	**0.63**	3.20
		Aqueous	8.00	6.00	8.00	12.00	8.00	8.00	NS	8.33
*Krauseola mosambicina* Caryophyllaceae (Moss.) Pax & K. Hoffm. (MNN-6)	Whole plant	Organic	4.80	7.20	6.80	7.20	8.60	5.50	1.40	5.93
		Aqueous	8.00	5.33	8.00	8.00	8.00	6.67	6.67	7.24
*Lippia javanica* Verbenaceae (Burm.f.) Spreng. (MNN-20)	Leaves	Organic	1.00	6.00	4.00	4.00	12.00	4.00	**0.50**	4.50
		Aqueous	8.00	4.00	6.00	8.00	4.00	6.00	8.00	6.33
*Mangifera indica* Anacardiaceae L. (MNN-29)	Leaves	Organic	**0.50**	**0.50**	1.00	1.00	**0.50**	2.00	**0.25**	**0.82**
		Aqueous	NS	6.67	2.67	NS	**0.50**	NS	4.00	3.46
*Melia azedarach* Meliaceae L. (MNN-4)	Leaves	Organic	2.89	1.16	1.70	4.28	1.81	2.64	**0.57**	2.15
		Aqueous	8.00	8.00	12.00	4.00	4.00	4.00	12.00	7.43
*Psidium guajava* Myrtaceae L. (MNN-5)	Leaves	Organic	**0.34**	**0.63**	**0.93**	1.63	1.51	**0.65**	**0.33**	**0.86**
		Aqueous	1.00	6.00	**0.50**	6.00	8.00	8.00	3.00	4.64
*Sarcostemma viminale* Apocynaceae (L) R. Br subsp. viminale (MNN-11)	Stem	Organic	1.00	8.00	4.00	16.00	16.00	2.00	**0.50**	6.79
		Aqueous	8.00	4.00	8.00	8.00	8.00	8.00	8.00	7.43
*Schotia brachypetala* Fabaceae Sond. (MNN-25)	Bark	Organic	8.00	**0.63**	2.00	8.00	1.50	8.00	**0.58**	4.10
		Aqueous	1.50	2.00	1.00	8.00	2.00	2.00	4.00	2.93
*Sclerocarya birrea* Anacardiaceae (A. Rich.) Hochst. subsp. caffra (Sond.) (MNN-12)	Bark	Organic	**0.29**	**0.29**	**0.35**	**0.95**	**0.75**	**0.20**	**0.34**	**0.45**
		Aqueous	2.00	2.00	**0.50**	2.00	4.00	1.33	2.00	1.98
*Senna occidentalis* Fabaceae (L.) Link (MNN-2)	Roots	Organic	3.00	3.00	2.00	1.00	3.00	2.00	4.00	2.57
		Aqueous	6.00	8.00	4.00	3.00	2.00	4.00	8.00	5.00
*Strychnos madagascariensis* Strychnaceae Pior. (MNN-9)	Leaves	Organic	3.00	4.00	2.88	4.00	3.25	4.75	1.00	3.27
		Aqueous	8.00	8.00	8.00	6.00	8.00	8.00	6.67	7.52
*Syzygium cordatum* Myrtaceae Hochst. ex. C. Krauss. (MNN-36)	Bark	Organic	**0.88**	1.82	1.00	1.81	1.25	1.54	**0.43**	1.25
		Aqueous	8.00	6.00	8.00	8.00	8.00	8.00	4.00	7.00
*Terminalia sericea* Combretaceae Burch. ex DC. (MNN-16)	Bark	Organic	**0.50**	**0.67**	**0.31**	1.00	**0.69**	**0.42**	**0.04**	**0.52**
		Aqueous	**0.38**	**0.50**	**0.75**	8.00	**0.50**	1.00	**0.67**	1.69
*Trichilia emetica* Meliaceae Vahl (MNN-35)	Bark	Organic	**0.25**	**0.16**	**0.25**	2.00	1.00	8.00	1.33	1.86
		Aqueous	6.00	8.00	4.00	4.00	4.00	12.00	4.00	5.00
*Vangueria infausta* Rubiaceae Burch. subsp. infausta (MNN-38)	Bark	Organic	8.00	3.25	1.00	8.00	6.00	3.50	1.25	4.43
		Aqueous	8.00	NS	8.00	8.00	4.00	8.00	8.00	7.33
*Vernonia natalensis* Asteraceae (DC) Sch. Bip. ex. Walp (MNN-33)	Roots	Organic	4.00	4.00	4.00	2.00	**0.25**	4.00	8.00	3.75
		Aqueous	2.00	6.00	8.00	6.00	1.50	8.00	6.00	5.36
Ciprofloxacin control (μg/mL)			0.50	0.25	0.80	0.02	0.40	0.20	0.05	0.32

MNN^a^ = MN Nkwanyana; NS^b^ = Not susceptible at highest concentration tested. Noteworthy values depicted in bold font.

In general, the organic extracts had better antibacterial activity than the aqueous extracts. This observation has been reported in a number of previous studies [23-25]. A study undertaken by Jäger [26], highlighted the poor activities of aqueous extracts in comparison with organic-derived extracts and raised concern in terms of antimicrobial efficacy when the traditional method is applied. It was thus interesting to see the superior efficacies found for the aqueous extracts of *A. burkei* (0.75 mg/mL) and *B. transvaalensis* (0.25 mg/mL) against *B. cereus.* These were three times and more than 32 times higher than the organic counterparts respectively. Furthermore, *B. transvaalensis* demonstrated efficacies eight times higher for aqueous extracts when tested against *S. flexneri.* Also, *Mangifera indica,* demonstrated noteworthy efficacies for the aqueous extract with a mean MIC value of 0.50 mg/mL against *P. vulgaris*.

*Terminalia sericea* was the only plant species to show broad-spectrum activity for the aqueous extracts having noteworthy activity against five of the seven pathogens studied (Table 1). The organic extracts showing the broadest spectrum of activity were *S. birrea* and *Garcinia livingstonei* (noteworthy activity against all pathogens and mean broad-spectrum MIC value of 0.45 mg/mL), followed by *T. sericea* (noteworthy activity against six of the seven pathogens tested with a mean broad-spectrum MIC value of 0.52 mg/mL). Other organic extracts demonstrating noteworthy broad-spectrum activity were *G. livingstonei, M. indica* and *P. guajava* (Table 1). The antimicrobial activity for *G. livingstonei* has been ascribed to the isolated compounds amentoflavone and 4”-methoxy amentoflavone which showed antibacterial activity against *Pseudomonas aeruginosa*, *Mycobacterium smegmatis, E. coli, S. aureus* and *E. faecalis* [27,28]. *Mangifera indica* is known as a traditional treatment for diarrhoea [2,29] and studies on *Shigella dysenteriae* have shown significant antimicrobial activity [30]. Further *in vivo* studies have shown that aqueous and alcoholic extracts of *M. indica* significantly reduced intestinal motility and faecal score in Swiss albino mice [31]. A review on *P. guajava* revealed that the aqueous and alcoholic extracts of this plant species possesses antimicrobial activity against a wide spectrum of pathogens [32].

*Garcinia livingstonei* was used moderately as an anti-diarrhoeal treatment by the lay people of northern Maputaland [7]. There is only one recorded use for *M. indica,* yet *P. guajava* was the most widely used (31 recordings for use as an antidiarrhoeal). This clearly indicates that there is not always a connection between high antimicrobial efficacy and frequency of use. To further substantiate this, *T. sericea,* demonstrating noteworthy efficacies for both aqueous and organic extracts is only moderately used as an antidiarrhoeal treatment [7].

The four combinations (Table 2) showed varying interactions towards different diarrhoeal pathogens. In some cases the interaction could not be determined (ND), as one or both plants had no end point MIC value. In these cases, comparison between efficacy of individual plant extracts and their combination resulted in a tentative interactive interpretation.

**Table 2:** **The mean MIC values (mg/mL) and ΣFIC values (given in brackets with interactive interpretation) of crude dichloromethane:methanol and aqueous extracts used in 1:1 combinations and tested against seven bacterial diarrhoeal pathogens**

**Plant combinations**	**Pathogens**															
	***B. cereus***		***E. faecalis***		***E. coli***		***P. vulgaris***		***S. typhimurium***		***S. flexneri***		***S. aureus***		**ΣFIC Averages**	
	**ATCC 11778**		**ATCC 29212**		**ATCC 8739**		**ATCC 33420**		**ATCC 14028**		**ATCC 25875**		**ATCC 12600**			
	**Organic**	**Aqueous**	**Organic**	**Aqueous**	**Organic**	**Aqueous**	**Organic**	**Aqueous**	**Organic**	**Aqueous**	**Organic**	**Aqueous**	**Organic**	**Aqueous**	**Organic**	**Aqueous**
*A. glabratum* and *K. mosambicina*	**0.03**	12.00	6.00	8.00	NS^a^	16.00	**0.16**	4.00	**0.63**	4.00	**0.03**	16.00	**0.04**	8.00	**0.30**	1.70
	**(0.02 S** ^**b**^ **)**	(1.88 NI^c^)	(1.13 NI)	(ND^d^ NI)	(ND ANT^e^)	(ND NI)	**(ND S)**	(N.D A^f^)	**(0.26 S)**	(0.63 A)	**(0.07 S)**	(3.20 NI)	**(0.01 S)**	(1.09 NI)		
*A. glabratum* and *P. guajava*	**0.03**	4.00	**0.31**	4.00	**0.16**	NS	**0.31**	2.00	**0.31**	8.00	**0.23**	8.00	**0.16**	2.00	**0.46**	2.00
	**(0.13 S)**	(2.38 NI)	(1.27 NI)	(ND S)	**(0.03 S)**	(ND NI)	**(ND S)**	(ND S)	**(0.37 S)**	(1.17 NI)	(0.78 A)	(2.33 NI)	**(0.17 S)**	(2.14 NI)		
*B. transvaalensis* and *P. guajava*	**0.02**	8.00	**0.16**	NS	**0.16**	16.00	**0.23**	2.00	**0.31**	2.00	**0.02**	8.00	**0.31**	4.00	**0.39**	2.33
	**(0.10 S)**	(ND NI)	(1.23 NI)	(ND NI)	**(0.06 S)**	(ND NI)	**(0.11 S)**	(ND S)	**(0.48 S)**	(ND S)	**(0.05 S)**	(2.33 NI)	(0.62 A)	(ND NI)		
*B. transvaalensis* and *S. birrea*	**0.03**	8.00	**0.63**	8.00	**0.63**	NS	**0.16**	3.00	**0.31**	12.00	**0.02**	1.50	**0.48**	3.00	0.88	0.57
	**(0.16 S)**	(ND S)	(3.32 NI)	(ND S)	**(0.34 S)**	(ND NI)	**(0.13 S)**	(ND A)	(1.10 NI)	(ND NI)	**(0.04 S)**	(0.57 A)	(1.11 NI)	(ND NI)		
Acetone control	16.00	16.00	NS	NS	NS	NS	13.30	13.30	NS	NS	8.00	8.00	NS	NS	NA^g^	
Ciprofloxacin control (μg/mL)	0.50		0.25		0.02		0.40		0.20		0.05		0.80			

NS^a^ = Not susceptible at highest concentration tested; S^b^ = Synergy; NI^c^ = Non interaction; ND^d^ = Not determined as no end point MIC is available for one or both plants. Tentative interaction is given based on MIC values. ANT^e^ = antagonism; A^f^ = Additive. NA^g^ = Not applicable; Noteworthy values depicted in bold font.

For the combination *A. glabratum* with *Krauseola mosambicina,* the 1:1 combination showed synergistic interactions against five of the seven pathogens studied (organic extracts) having a mean (across all pathogens) ΣFIC value of 0.30. The most significant interaction was against *S. aureus* where MIC values for individual organic plant extracts were 8.00 mg/mL. When combined, a 200 fold increase in activity (MIC 0.04 mg/mL and ΣFIC 0.01) was noted. The combination of *A. glabratum* with *K. mosambicina* is also the most widely used combination by the residents of the homesteads from northern Maputaland [7]. The combination of *A. glabratum* with *P. guajava* demonstrated five and two synergistic interactions for the organic and aqueous extracts respectively with a mean (across all pathogens) ΣFIC value of 0.46. The most significant interaction with this plant combination was against *E. coli* where MIC values for individual organic plant extracts were 12.00 mg/mL and 4.00 mg/mL respectively. When combined, at least a 25 fold increase in activity (MIC 0.16 mg/mL and ΣFIC 0.03) was noted. *Brachylaena transvaalensis* and *P. guajava* (organic extracts) were mostly synergistic (mean ΣFIC value of 0.39) when combined. The highest activities were observed against *E. coli* and *S. flexneri* where the combination had at least a 19 fold increase in activity. The most synergistic interaction for the combination *B. transvaalensis: S. birrea* was against *S. flexneri* with an ΣFIC of 0.04. Only one tentative antagonistic interaction was observed for the organic extracts of *A. glabratum* combined with *K. mosambicina* when tested against *E. coli.* All organic extract combinations demonstrated synergy against *B. cereus* and *P. vulgaris*. Both these pathogens are strongly linked to diarrhoeal diseases and thus demonstrate some validity to the selection of these plants to treat such infections. The homestead residents use the combinations in aqueous form but the tests showed mainly non-interactive interactions. Possibly an *in vivo* screening approach might yield different outcomes to the *in vitro* testing observed here. Furthermore the practitioners may be using the combined plants for relief of other symptoms (e.g. antispasmodic, anti-inflammatory effects) and not merely as an antimicrobial.

## Conclusions

Plants collected from the homesteads in Maputaland are a sustainable way of harvesting and managing medicinal resources. The traditional use of the selection of plants, as presented here, for the treatment of stomach ailments provides some insight into bacterial efficacy. Selected plants (*G. livingstonei, M. indica, P. guajava*, *S. birrea* and *T. sericea*) used individually show broad-spectrum activity yet only *P. guajava* and *S. birrea* are frequently used*.* This study also provides some insight into the neglected area of *in vitro* efficacy testing of plant combinations as an anti-diarrhoeal treatment. Plant combinations demonstrated favourable efficacy with mostly synergistic effects noted, lending some credibility to their use in combination. Finally, it should be noted that while bacterial enteropathogens, as tested herein, are commonly associated with diarrhoea, other pathogens such as the rotavirus and parasites such as *Entamoeba histolytica* may also contribute toward the burden of diarrhoeal diseases and as such, it is recommended that further investigations of these plants should be undertaken on these neglected pathogens.

## Abbreviations

MIC: Minimum inhibitory concentration
ΣFIC: The sum of the fractional inhibitory concentration also known as the fractional inhibitory index
